# Successful Management of a Patient With a History of Postoperative Delirium Undergoing Cardiac Surgery With an Erector Spinae Plane Block and Multimodal Analgesia: A Case Report

**DOI:** 10.7759/cureus.25504

**Published:** 2022-05-30

**Authors:** Michael Hsu, Sudhakar Kinthala, Jordan Huang, Jaimi Philip, Poovendran Saththasivam, Burdett Porter

**Affiliations:** 1 Anesthesia, Guthrie Robert Packer Hospital, Sayre, USA

**Keywords:** opioid-free general anesthesia, opioid-free analgesia, eras protocol, erector spinae plane (esp) block, postoperative delirium

## Abstract

Perioperative delirium is an acute confusional state with fluctuating levels of consciousness, which can be precipitated by opioid-based anesthetics and inadequate pain control, especially in patients undergoing cardiac surgery. We seek to minimize opioid usage to avoid postoperative delirium in a patient with multiple risk factors undergoing aortic valve replacement. We used cardiac enhanced recovery after surgery protocols (ERAS-C), which include multimodal analgesia and regional anesthesia via bilateral erector spinae plane (ESP) blocks. Our observations suggest that bilateral ESP blocks and cardiac ERAS protocols offer a potential option to manage pain and control risk factors in patients at high risk of postoperative delirium undergoing cardiac surgery.

## Introduction

Opioid-based anesthesia was previously considered the gold standard for open cardiac surgery because of better hemodynamic stability and postoperative pain control despite complications such as respiratory depression, nausea, vomiting, and hyperalgesia [1]. Cardiac enhanced recovery after surgery protocols (ERAS-C) include multimodal analgesia and regional anesthesia and are utilized to decrease opioid requirements to accelerate recovery and shorten the postoperative length of stay [2]. There is a growing consensus that using opioids for anesthesia can precipitate perioperative delirium [3]. Here, we present a case of successful implementation of ERAS-C, erector spinae plane block, and multimodal analgesia to mitigate the occurrence of perioperative delirium in a patient with multiple risk factors undergoing aortic valve replacement.

The Diagnostic and Statistical Manual of Mental Disorders, Fifth Edition (DSM-5) describes delirium as an acute change in attention and awareness that develops over a relatively short time interval [4] and is associated with additional cognitive deficits such as memory deficit, disorientation, or perceptual disturbances [5]. It is an acute confusional state that has a wide spectrum of effects on the patient and the healthcare system. Patients with delirium have prolonged neuropsychological dysfunction, high hospital morbidity and mortality, increased healthcare costs, and longer lengths of stay in the intensive care unit (ICU) [6-7]. The etiology of perioperative delirium is multifactorial. A few precipitating factors are undergoing cardiac surgery, administration of benzodiazepines, pain, hypoxia, and opioid-based and general anesthesia [5]. Delirium is reported in approximately 50% of patients, and increased levels of postoperative pain were independently associated with a greater risk of postoperative delirium [8].

Given that opioid-based anesthetic approaches can precipitate delirium, previous studies have sought to minimize opioid administration to mitigate associated complications [9]. We describe an opioid minimization technique in a patient with multiple risk factors for postoperative delirium undergoing open aortic valve replacement (AVR) using multimodal analgesia and bilateral erector spinae plane (ESP) catheters to provide proper pain control.

The patient provided written permission for the publication of this case report and the manuscript adheres to CARE (CAse RE) guidelines.

## Case presentation

We present the case of a 79-year-old, 88-kg Caucasian man with a body mass index of 27.91 kg/m^2^ and body surface area of 2.06 m^2^ with severe symptomatic aortic regurgitation requiring aortic valve replacement (AVR) surgery. This patient had a history of dyspnea on exertion, progressive memory issues, and cognitive dysfunction, and hence was classified as American Society of Anesthesiologists (ASA) grade 4 for pre-anesthesia comorbidities. Preoperative echocardiography demonstrated severe aortic insufficiency with prominent holodiastolic flow reversal and moderate-to-severe concentric left ventricular hypertrophy with normal LV systolic function. Preoperative complete blood count, basic metabolic panel, and albumin levels were within normal limits, except for elevated blood urea nitrogen (BUN) (24 mg/dl) and creatinine (Cr) (1.2 mg/dl). The patient had a history of opioid-induced delirium following cardiac catheterization done under monitored anesthesia care, resulting in a prolonged post-procedure hospital stay. The patient’s advanced age and significant delirium following cardiac catheterization indicated that postoperative delirium may also occur after the AVR. Acknowledging the patient’s and his family’s requests, we performed the surgery with the opioid minimization strategy.

**Figure 1:**
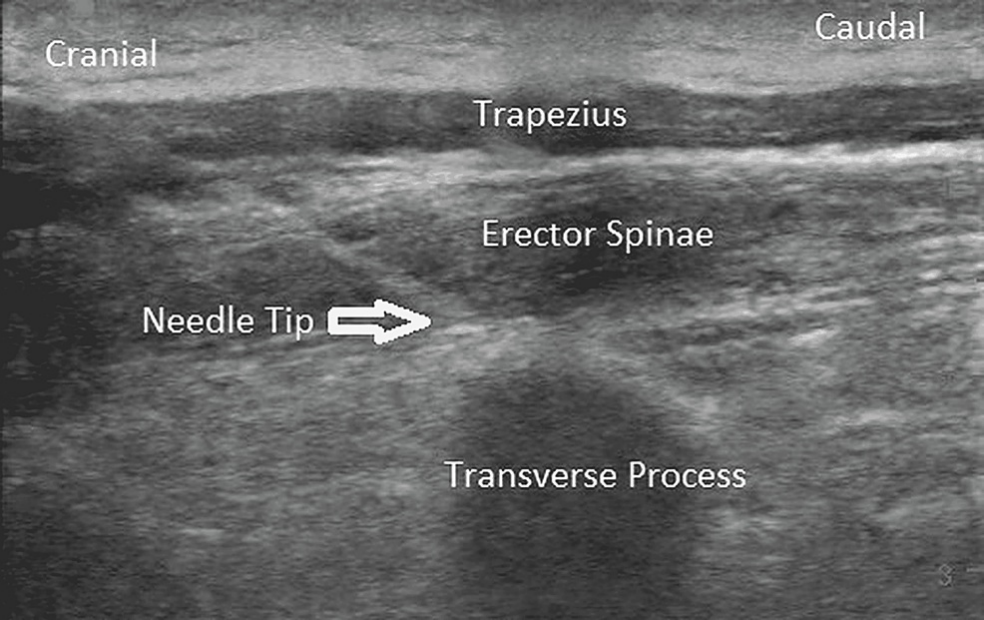
Erector spinae plane under longitudinal parasagittal ultrasound visualization; needle tip in a plane deep to the erector spinae muscle

**Figure 2:**
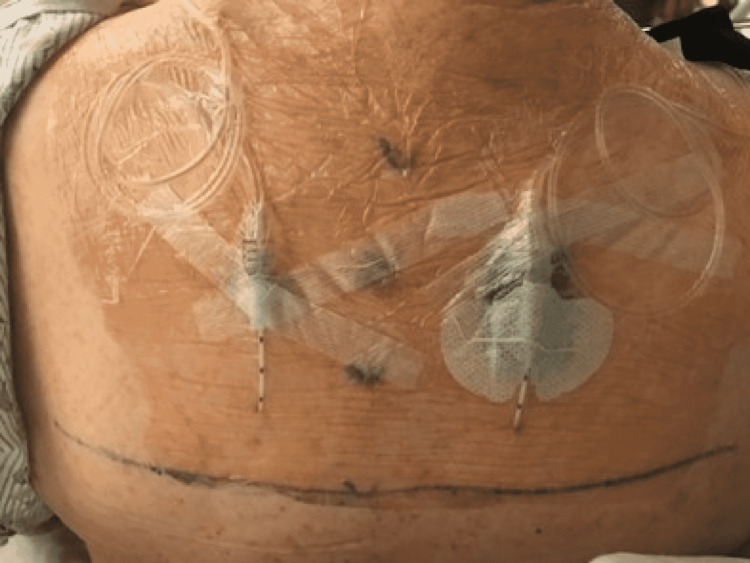
Bilateral erector spinae plane catheters at the thoracic vertebra 5 level secured on the back of the patient.

Bilateral ESP catheters were placed at the thoracic vertebra 5 (T5) level deep to the erector spinae muscle (Figures 1-2) under ultrasound guidance in the interfascial plane. Hydro-dissection was used to create a space to allow the insertion of the catheter. A 25-mL bolus of 0.5% ropivacaine was administered through each ESP catheter, and a multimodal analgesia regimen consisting of 1 g oral acetaminophen, 300 mg oral gabapentin, and 200 mg oral celecoxib was administered.

Standard ASA monitors, an arterial line, and a pulmonary artery catheter were placed for open AVR. We avoided benzodiazepines and ketamine to minimize the chances of postoperative delirium. Otherwise, intraoperative anesthesia management was performed in a standard fashion with 2.2 mg/kg intravenous propofol for induction and 1.2 mg/kg rocuronium or neuromuscular blockade. Anesthesia was maintained with 0.7 L/min oxygen, 1.3 L/min air, and 1% isoflurane. Prior to the surgical incision, intravenous dexmedetomidine infusion was initiated at 0.7 mcg/kg/h to maintain an intraoperative bispectral index spectrometry level of less than 60. An additional 25 mL of 0.5% ropivacaine was infused through each ESP catheter 4 h after the initial dose. Heart rate and blood pressure were maintained within 20% of the preoperative values without requiring intraoperative opioid pain medication.

Using this multimodal schedule, hemodynamic stability was achieved, and there were no intraoperative surgical or anesthesia critical events. Postoperatively, the patient was transferred to the ICU and successfully extubated 4 h later. The pain was controlled with intermittent boluses containing 15 mL of 0.5% ropivacaine every 3 h through each ESP catheter and 1 g PO acetaminophen every 8 h. Gabapentin and celecoxib were not continued postoperatively. The patient could easily draw 1750-2200 cc (19 cc/kg - 25 cc/kg) on incentive spirometry on postoperative day 0, and by postoperative day 1, he was ambulating and out of bed without difficulty. Patient visual analog scale (VAS) pain scores were consistently less than 3 postoperatively and no scheduled or rescue opioid analgesia was required. The patient had an unremarkable hospital recovery course and was discharged on postoperative day 4. No clinical features of delirium or cognitive dysfunction were noted during the office follow-up visits.

## Discussion

In this case report, we sought to minimize opioids and provide an appropriate anesthetic technique for a 79-year-old male with a history of postoperative delirium undergoing cardiac surgery. We found that the patient’s hospital recovery following the surgery was uneventful and no evidence of postoperative delirium was acknowledged during follow-up.

Regional anesthesia has become an integral component in reducing opioid requirements during cardiac procedures. Investigators have reported that its use can also provide better pain control, reduce opioid consumption, and improve extubation time compared to the use of IV medications [10]. In 2016, the ESP block was introduced as an injection of local anesthetic 3 cm away from the thoracic transverse process deep into the erector spinae muscles. The developed plane allows for the spread of local anesthetic anteriorly into the paravertebral and epidural spaces to block the dorsal and ventral rami of the thoracic spinal nerves [11]. In comparison to thoracic epidural analgesia, it has been found that the ESP block achieved a comparable VAS score, incentive spirometry, time on the ventilator, and ICU duration without the potential risk of expanding epidural hematoma after heparinization, accidental dural puncture, and neurologic injury [11]. ERAS minimization of opioids with adjunctive ESP catheters has been studied for effective pain management following cardiac and thoracic surgery, with ESP catheters decreasing 48-hour opioid consumption by almost 30% [10]. It has been shown that ESP catheters alone provide more robust and longer-lasting pain relief compared to IV acetaminophen and Toradol [12]. Additionally, postoperative adverse events, such as episodes of hypotension, nausea, vomiting, and hyperglycemia, were markedly decreased with ESP catheters [13]. Chanowski et al. are among the reported cases where the successful use of an ESP catheter demonstrated an opioid-free, ultra-fast-track coronary artery bypass graft procedure without postoperative complications [14]. Early studies have demonstrated that it is a safe alternative to epidural anesthesia for video-assisted thorascopic surgery, and when compared to regular bupivacaine, the liposomal formula can also result in reduced opioid consumption [15].

Our patient had multiple risk factors for postoperative delirium: advanced age, cognitive dysfunction and memory loss at baseline, and a history of post-procedure opioid-induced delirium. In this case, we minimized the usage of opioids and avoided benzodiazepines and ketamine, which are precipitating factors for postoperative delirium. Instead, we used a multimodal analgesia protocol consisting of oral acetaminophen, gabapentin, celecoxib, dexmedetomidine infusion, and placement of bilateral ESP catheters, which are used on a regular basis with proven benefits [10]. Preoperative acetaminophen, gabapentin, and intraoperative dexmedetomidine infusions are used in multimodal analgesia and cardiac ERAS, and their effects are also well-documented [9,16].

The dexmedetomidine infusion has been found in cardiac surgery to significantly reduce the duration of delirium [17] and achieve targeted sedation and adequate analgesia compared to the morphine regimen [18]. However, there is a recent controversy over dexmedetomidine’s ability to decrease delirium in cardiac surgery and recent studies have shown evidence contrary to that [19]. Kharasch et al. highlighted a recent clinical trial that was stopped prematurely over dexmedetomidine safety concerns of severe bradycardia and a higher incidence of hypoxemia [20].

## Conclusions

In the present case, with multiple risk factors for postoperative delirium, providing adequate pain relief and avoiding opioids could have significant implications for mitigating postoperative delirium. Moreover, the use of ropivacaine and multimodal analgesia reduced opioid use. We believe that bilateral ESP pain catheters reduced the controllable risk factors for postoperative delirium and minimized opioid administration to prevent postoperative delirium. Our observations indicate that bilateral ESP catheters and multimodal analgesia mitigate the risk factors and can potentially prevent postoperative delirium in high-risk patients undergoing cardiac surgery.
